# Knee Measurement System with Osteoarthritis Levels Using Artificial Cartilage and Skeletons

**DOI:** 10.3390/biomimetics9030166

**Published:** 2024-03-08

**Authors:** Minchae Kang, Suyeon Seo, Hyewon Lee, Min-Woo Han

**Affiliations:** 1 Department of Mechanical Engineering, Advanced Manufacturing & Soft Robotics Lab, Dongguk University, 30 Pildong-ro 1, Jung-gu, Seoul 04620, Republic of Korea; trishakang@dgu.ac.kr (M.K.); tndus7058@dgu.ac.kr (S.S.); 2 Department of Mechanical, Robotics and Energy Engineering, Advanced Manufacturing & Soft Robotics Lab, Dongguk University, 30 Pildong-ro 1, Jung-gu, Seoul 04620, Republic of Korea; lhw2202@dgu.ac.kr

**Keywords:** 3D printing, soft polymer, artificial joint, mimicking knee movement, four-bar-link theory, surgical simulator, Kellgren–Lawrence grade

## Abstract

Knee osteoarthritis (OA), also known as degenerative arthritis, is a disease characterized by irreversible changes in the cartilage and bones comprising the joints, resulting in pain, impaired function, and deformity. Furthermore, independent of natural aging, the rate of change in joint cartilage has increased in recent years, which is mainly attributed to environmental factors. The rising incidence of knee-related disorders emphasizes the importance of analyzing the morphology and kinematics of knee structure. This study introduces a knee measurement system designed to replicate the motions of knee using 3D-printing technology, providing insights into knee mechanics with OA level. The research explores the stages of OA using the Kellgren–Lawrence (KL) grade scale, highlighting the variations in the force applied to the knee bone according to movement. The developed knee-simulation system, utilizing the four-bar-link theory, presents a novel approach to studying OA levels 0 to 4. As OA progresses, the cartilage deteriorates, affecting the movement of OA. The OA-based knee measurement system that incorporates soft tissues and skeletons can assist in developing a personalized diagnostic approach for knee disease. This will also help to enhance surgical effectiveness by facilitating the creation of personalized prosthetic joints for individual patients and offering a customized surgical simulation.

## 1. Introduction

Osteoarthritis (OA) is a disease that damage bones and ligaments by slowly destroying cartilage. This causes pain and inflammation [1,2,3]. The number of osteoarthritis patients worldwide has continued to increase since 1990 and is expected to reach 1 billion by 2050 [4]. The prevalence of OA is a significant public health concern, as it not only impacts the quality of life for millions of individuals, but also places a considerable burden on healthcare systems. The statistics emphasize the urgency of developing advanced tools and technologies to better understand, diagnose, and manage conditions like OA.

In light of the rising incidence of OA and other knee-related disorders affecting millions annually [4], there is a critical need for a sophisticated knee measurement system that mirrors the intricate movements of the human knee and can accurately measure the forces acting on the femur. Traditional assessment methods often lack the precision required to comprehensively analyze the dynamic biomechanics of the knee joint. The knee measurement system addresses this gap by providing a simulation of human knee movement and offers invaluable insights into the mechanics of knee disorders. Furthermore, the capacity to quantify the force exerted on the femur during motion is critical to comprehending the distribution of stress in the joint, supporting the early identification of abnormalities and enabling customized treatment plans. By integrating such a system into clinical practice, we not only enhance diagnostic accuracy, but also pave the way for personalized and effective interventions, ultimately improving the overall management of knee-related conditions and enhancing patient outcomes.

**Figure 1:**
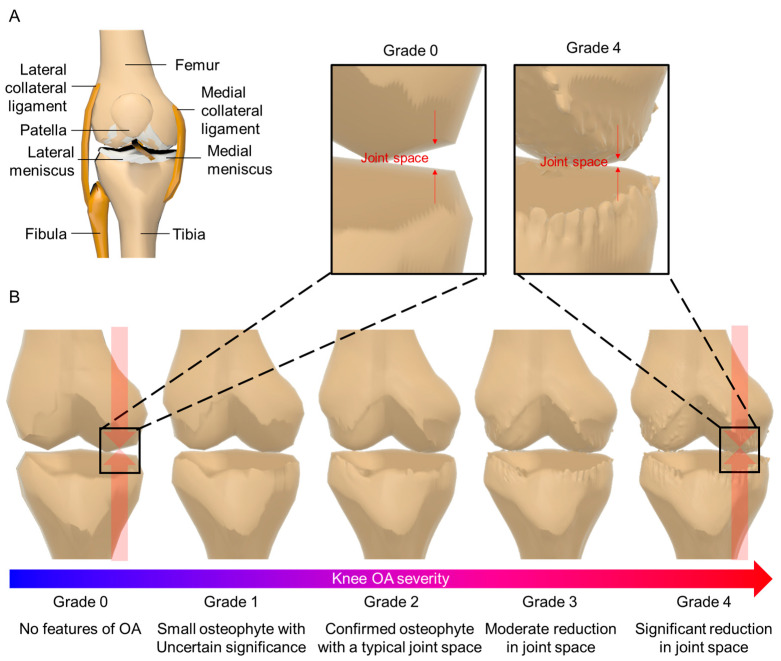
(**A**) Shows the structure of the knee bone, and (**B**) shows the shape of the knee bone varies according to the KL grading scale and the detailed difference between Grade 0 and Grade 4.

The knee bone consists of the femur, tibia, and fibula (Figure 1A). As osteoarthritis (OA) progresses, osteoclasts form and bony protrusions develop (Figure 1B). Cartilage wears out, and the joint space narrows. OA severity is classified into levels 1 to 4 using the Kellgren–Lawrence (KL) grade scale based on radiographic findings and symptom progression [5,6,7]. Here, level 0 means normal knee. Symptoms, x-rays, and MRI images are used to define OA levels. Grade 1 shows a slight bone gap, suggesting possible OA. Grade 2 presents a clear bone gap, but the gap between the knee joints is not significantly narrowed. In Grade 3, the gap starts to narrow. Finally, Grade 4 exhibits a significantly reduced gap and visible bone spurs (Figure 1B).

Several studies have explored the relationship between patients’ OA Levels and changes in force applied to cartilage and ligaments due to variations in the knee joint space, both in magnitude and direction [8,9,10]. Subsequently, researchers have investigated the impact of each patient’s specific knee bone shape on bone pressure and skeletal movement [11,12,13,14,15,16].

Research in knee simulation is actively progressing. Shriram et al. developed a 3D-based finite element (FE) model of the human knee joint. They also simulated an anatomically formed arbitrary meniscal implant, and it’s stiffness was 11 MPa, 15 MPa, and 17 MPa [17]. As the stiffness of the implant material increases, in the cartilage, contact pressures, compression stresses, shear stresses, and compressive strains increase, but the implant displacement decreases. In the Open Knee project, a shape of the lower knee structure was fabricated. It was extracted from a magnetic resistance (MR) image of a cadaveric knee sample of a 70-year-old woman. The Open Knee provides a computational representation of the anatomy and dynamics of the tibia femoral joint [18]. As a result of the simulation of meniscectomy, it was confirmed that the internal and external compression of the tibia cartilage engaged with the femoral cartilage increased compared to the use of a complete meniscus.

The force applied to the knee depends on the movement of the joint. In the study of Kutzner et al., the average peak of result force was the highest at 346% body weight (BW) when going down the stairs after checking the knee joint load on four male and one female subjects [19]. Subsequently, when going up the stairs (316% BW), level walking (21% BW), one registered state (259% BW), knee bending (253% BW), standing up (246% BW), sitting down (225% BW), and two registered state (107% BW), the average peak of the resulting force decreases in order. Also, according to Masouros et al., a maximum knee flexion of 100° is required to straighten the body when standing up from a chair [20]. Theoretically, when standing up from a chair, patellofemoral force (550% BW) and tibiofemoral force (350% BW) are applied. It was confirmed that the peak shear force was 10–20 times smaller than the axial force. In addition, according to Bendjaballah et al., when the knee is in a 5° valgus position, the force applied to the interior cruciate ligament (ACL) becomes six times stronger than when it is the front plane [21].

Research related to the mimicking structure and shape of the knee is continuing. Etoundi et al. present a bio-inspired condylar prosthetic knee joint, offering a mechanically advantageous design over traditional hinge joints [22]. Bian et al. focus on morphological characteristics of cartilage-bone transitional structures in the human knee, leading to the CAD design of a biomimetic osteochondral scaffold [23]. This scaffold, mimicking native bone structure, addresses current tissue engineering limitations and shows potential for creating an osteochondral complex. Wang et al. presents a dual-layer biomimetic cartilage scaffold for knee OA repair [24]. The scaffold, with orientated porous structures, effectively guides bone marrow stem cells, demonstrating promising results in a rabbit model. This work has implications for developing multilayer scaffolds resembling articular cartilage’s zonal organization. Fisher et al. developed organized nanofibrous scaffolds to mimic the knee meniscus’s macroscopic and microscopic architecture [25]. These circumferentially aligned nanofiber scaffolds show potential for creating functional anatomic meniscus constructs, addressing challenges in tissue engineering for knee joint load transmission.

The knee-joint robot developed by Morita is made to be three-quarters the size of a standard adult male and simulates the left leg. It consists of the femur, tibia, and knee joints. It is a 2-degree-of-freedom robot with an extension range of 0°, a flexion range of 140°, and a rotation range of 10°, which implements rotational motion through three rods and bearings. The Knee-joint robot may mimic problems of knee joints such as range of motion tract, contraception, rigor, and spasticity [18]. Externally powered above knee prosthesis (AKP) is a motor and controls flexion and extension movement of artificial knee-joint. This analyzes walking patterns and implements lower body exercises such as sitting, standing, walking backward, and walking stairs using computer codes. For implementation, marks are placed on the applicant’s toes, 5th metatarsal, heels, ankles, knees, hips, and ribs. As a result, the peaks of the knee flexion angle are 15.7°, 7.2°, 60.5°, and 0°.

Since the human knee has complex behavior, it is difficult to implement it through simple axial mechanical behavior. Therefore, various methods have been proposed as in the following studies [26]. The oxford simulator is based on Bourne and Goodfellow [27] at Oxford University, allowing for six degrees of freedom in knee kinematics while adjusting the quadriceps load to induce knee flexion/extension. This design has been the basis for various knee simulators [28,29]. And some robotic systems were also proposed [30,31]. As a method for this, a four-bar-link theory was proposed.

The four-bar-link theory is a method used to facilitate the movement of the knee rotation axis, including rolling and sliding mechanisms. The four-bar-link may be used as four links together with two bones, the tibia and the femur, using the anterior and posterior cruciate ligaments as the respective links to realize the movement of the knee. Exceptional efficiency can be attained by utilizing lightweight, cost-effective links with less friction [32]. However, there is an inductive error in link design. Therefore, mechanical advertisements, ranges, dead-center positions, fabrication, and backlash should be considered for a successful design [33]. The four-bar mechanism can also be employed to produce particular trajectories of mechanical constructions [34]. To represent the path of the desired point, the discrete path is drawn using finite numerals of points. After that, the coupler link may be located [35]. Natural movements may be implemented by using four-bar mechanism. As an example, Xydas et al. developed a mechanism that mimics natural hand movements using motors, springs, and links and applied it to rehabilitation robots [36]. In addition, Yoon produced a robot finger of three DOFs using one linear actuator and two springs [37]. González significantly reduced energy consumption by optimally designing four-bar linkage on the suspension of the off-road racing bicycle [38].

In this work, through the four-bar-link theory, a knee simulation system was designed and manufactured with 3D printing technology. Corresponding to each level of OA, cartilage was created using a soft polymer and attached to respective tibias. The tibia segment was affixed to a load cell, while the femur was driven by a motor, facilitating the measurement of force changes to confirm their variation according to OA levels. Investigating the force magnitude and direction applied to cartilage and ligaments across OA levels 0 to 4 revealed distinct characteristics at each stage. This study examined the traits of OA levels 0 to 4 by measuring the force applied to the femur bone, providing valuable insights into the knee bone’s condition during simulated patient exercises resembling real walking scenarios. The developed measurement system, assessing force under the femur bone, is instrumental for analyzing differences among OA patients, and can be applied in various fields, including the advancement of surgical simulators.

## 2. Materials and Methods

**Figure 2:**
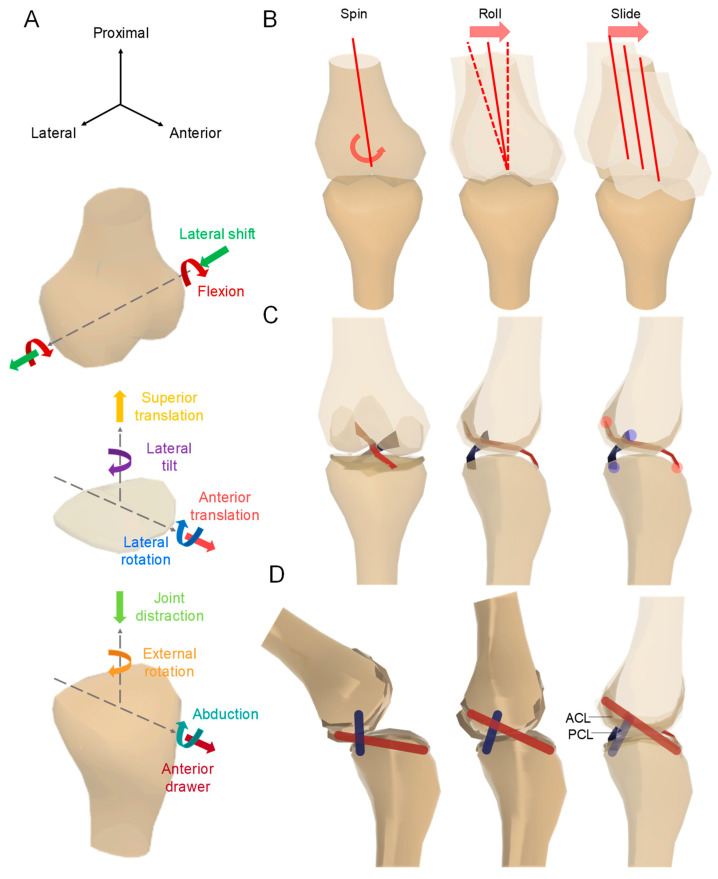
(**A**) Shows the axis of the knee joint. (**B**) Shows the spin, roll, and slide movements of the tibia (femur); (**C**,**D**) show the knee movement of the four-bar-link.

Knee movement is not a simple rotational exercise, but a six-degree of freedom, including a rolling and a sliding, motion about the rotational axis as shown in Figure 2A,B. Accordingly, the rotational axis of the knee continuously changes according to the movement. The continuous changes in the rotational axis ensure that the knee joint can effectively distribute forces, absorb shocks, and maintain stability throughout a range of movements. The coordination between muscles, ligaments, and the joint structure allows for this dynamic adjustment, highlighting the remarkable biomechanical sophistication of the human knee. Understanding the continuous changes in the rotational axis of the knee is fundamental in fields such as biomechanics, orthopedics, and sports science. It provides insights into the complexities of human movement, aiding in the development of interventions, treatments, and preventive measures for conditions related to the knee joint. Spinning, rolling, and sliding are the three movements of the knee (Figure 2B). The four-bar-link theory is used to implement rolling and sliding during this knee movement.

The four-bar-link is a concise model for implementing the complex biomechanics of the knee joint. The knee joint is an intricate structure and is composed of a number of ligaments, cartilage, and muscles that enable various movements, but four-bar-link simplifies this complexity.

The theory using four-bar-link is the implementation of knee motion with four points and two links. As shown in Figure 2C, four-bar-link is a method that assumes one link each of the anterior cruciate ligament (ACL) and posterior cruciate ligament (PCL), which correspond to the cruciate ligament. Two ligaments and two bones become links to form a four bar. At both ends of the ligament of the knee bone model, the link connecting position penetrates the bone and it becomes four-bar-link (Figure 2D). This work utilized the four-bar-link theory to simulate knee movement in a fabricated knee model.

**Figure 3:**
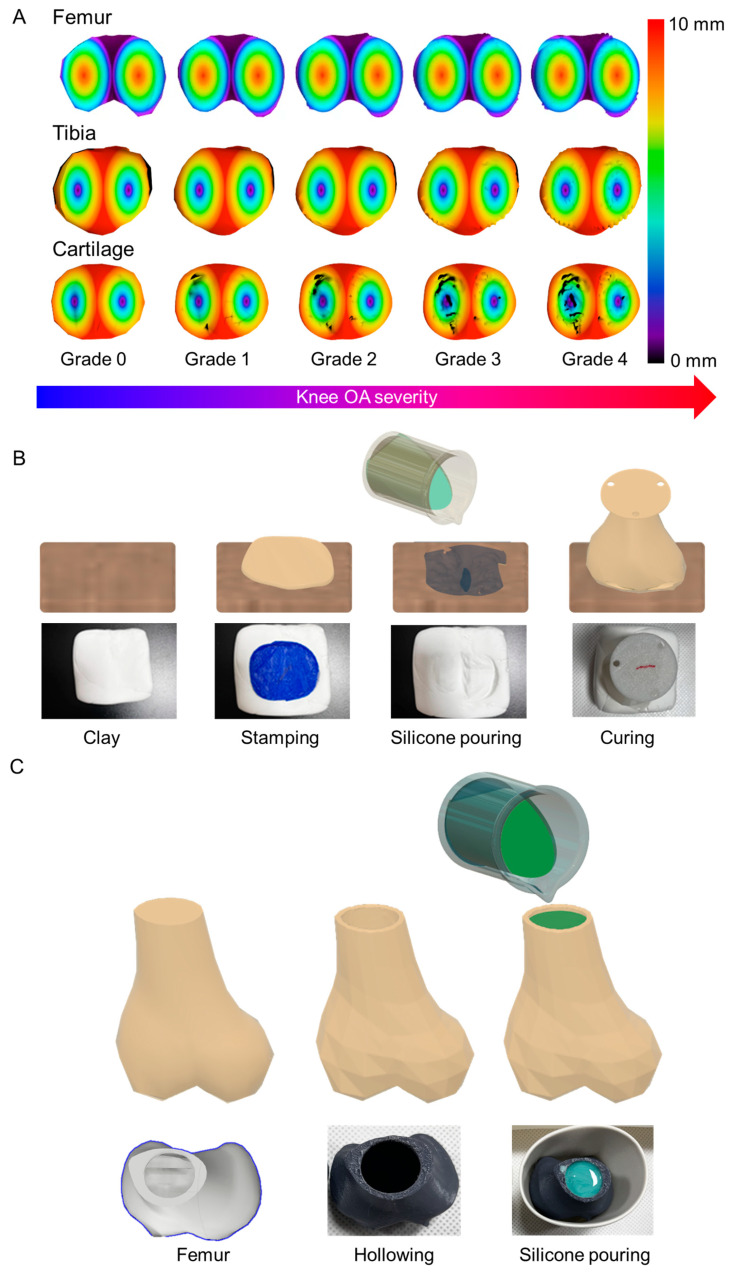
(**A**) 3D model of bone used in the study. Femur, tibia, and cartilage in a row according to the OA level. (**B**) The cartilage production method. (**C**) Shows the manufacturing steps of a 3D-printed exoskeleton with polymer filling inside.

3D model of the knee bone is shown in Figure 3A. Figure 3A demonstrates a correlation between increasing OA levels and the erosion of femur and tibia bones, accompanied by a reduction in cartilage thickness and an increase in its roughness. The color bar on the *y*-axis indicates depth, with the maximum depth set at 10 mm. By examining the height variations in each bone, it becomes apparent that as the severity of the disease increases, there is significant flexion and a corresponding increase in the amount of cartilage that is removed. As the level increases, the thickness of the cartilage becomes thinner and uneven. The specific design of the knee bone model was redrawn through 3D modeling programs after converting patient CT data into 3D models using the InVesalius program. Based on this, it was printed in an FDM method using Polyactic acid (PLA) filaments through a 3D printer, followed by a detailed fabrication method. PLA and human knee components’ material properties are compared in Table 1.

The cartilage was attached on the printed bones. An artificial femur is required as a first step in fabricating the cartilage. According to the KL scale, it was printed by applying the four-bar-link insertion position corresponding to Grade 1–4. In order to apply the soft properties of cartilage to artificial bones, the shape of cartilage was cast and attached to the tibia in the form of polymer. The shape and thickness of cartilage, which differ for each patient with disease, were designed based on CT, and were manufactured by 3D printing. After manufacturing the shape by placing the printed cartilage on the clay, ecoflex is poured. Before the polymer hardens, the 3D-printed femur is placed on it to make the structure of it. After hardening, clay was removed to complete the femur. The fabrication process of the artificial cartilages is shown in Figure 3B.

**Figure 4:**
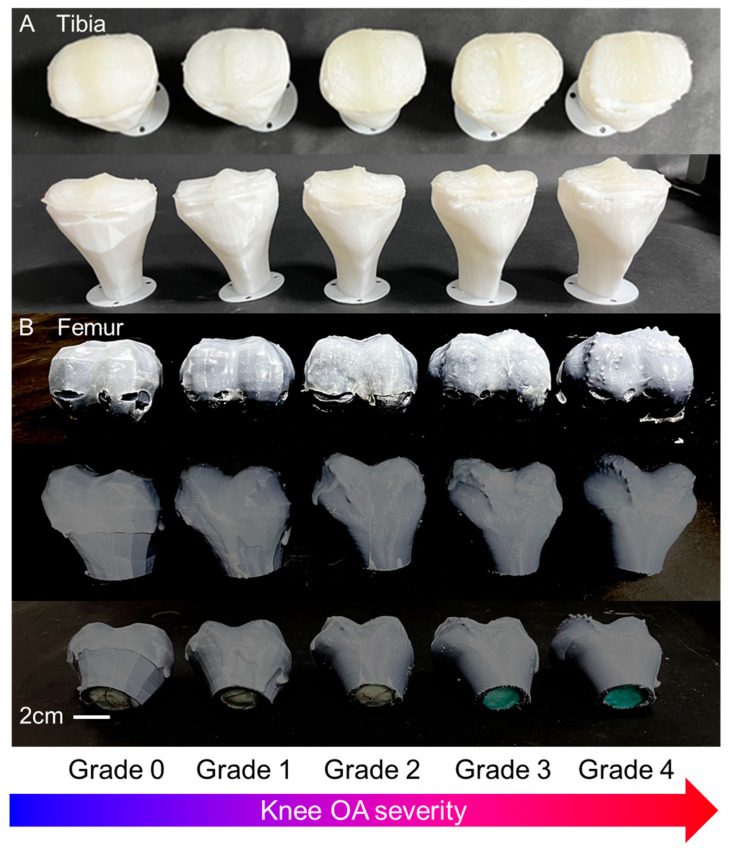
(**A**) The3D-printed tibias and (**B**) fabricated femurs.

The next step is to manufacture the tibia. The tibia was printed with a 3D printer by combining a plate that could be secured after cutting a certain point of the tibia so that it could be attached to the load cell. The method for cartilage production is the same as that of the femur. Furthermore, a polymer was infused into the interior of the 3D printed bone to achieve a similar weight to that of natural human bone. In order to accomplish this, the interior of the printed bone was hollowed out, and a polymer (Smooth-Sil 933, Smooth-On, Inc, Pennsylvania, United States.) was introduced into the void and subsequently solidified (Figure 3C). The specific gravity of Smooth-Sil is 1.42 g/cc and the specific gravity of PLA is 1.24 g/cc [39,40]. The results of attaching the casted cartilage to the tibia and femur are shown in Figure 4.

**Figure 5:**
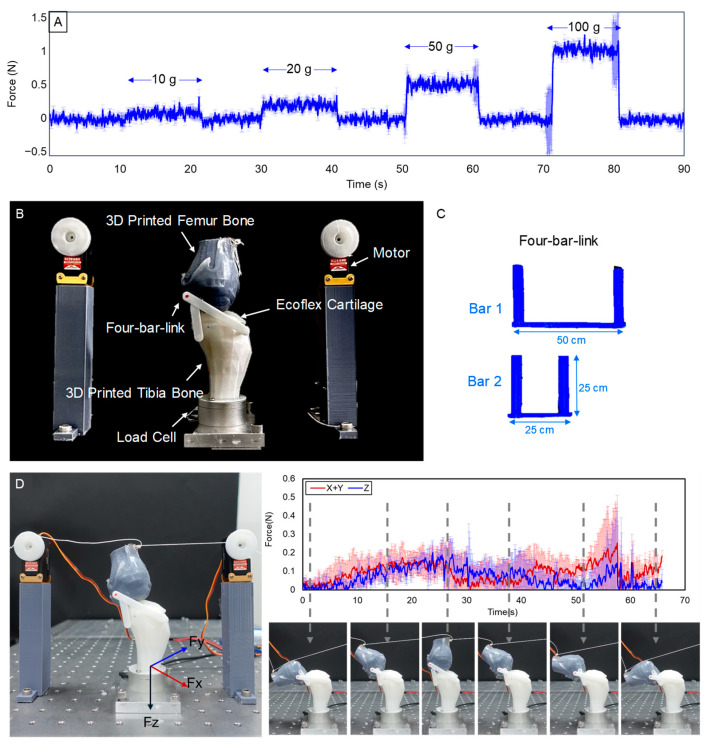
(**A**) Shows the experiment weights of 10 g, 20 g, 50 g, and 100 g applied to the force measurement sensor, (**B**) shows the experiment set-up to measure the force applied to the femur, (**C**) shows the fabricated four-bar-link, and (**D**) is the force measurement for the normal knee bone movement.

To evaluate the characteristics of knee movement using the artificial model, the cartilage-mounted artificial tibia is secured with a force measurement sensor, and the tibia and femur are connected by a link. The rated capacity of the force measurement sensor used in this experiment is force 100 N, torque 2.5 N·m, and the resolution is force 0.2 N, torque 0.0008 N·m. In addition, before measuring the force applied to the knee, an accuracy evaluation experiment was conducted. This experiment was conducted by raising and lowering the weights of 10 g, 20 g, 50 g, and 100 g every 10 s. As a result, a slight error occurred, but it was confirmed that the weight was well reflected in the value (Figure 5A). The experimental setup is shown in Figure 5B,C shows the fabricated four-bar-link. Force was measured according to the joint movement of the cartilage attached model. The force measurement sensor under the tibia captures the forces exerted through the tibia during motions. This could include walking, running, or other activities that mimic real-life scenarios. The sensor measures forces in two dimensions which are axial and anterior. A thread was connected to the upper fixture of the artificial bone femur and pulled by a motor to cause a knee bending motion. At this time, the motor for adjusting the femur was aligned with the end of the knee bone femur. While using the thread, tension force was included when the motor location was lower than the femur. It could be solved using the motor in the same position as the end of the femur. The amount of force change in the x, y, and z axes was measured using a force measurement sensor fixed to the lower end of the tibia. The same experiment was conducted on the normal model and OA Levels 1–4. Figure 5D represents the force measurements of the load cell in relation to the direction of force during normal movement of the knee bone.

## 3. Results

### 3.1. Force Measurement According to OA Level

**Figure 6:**
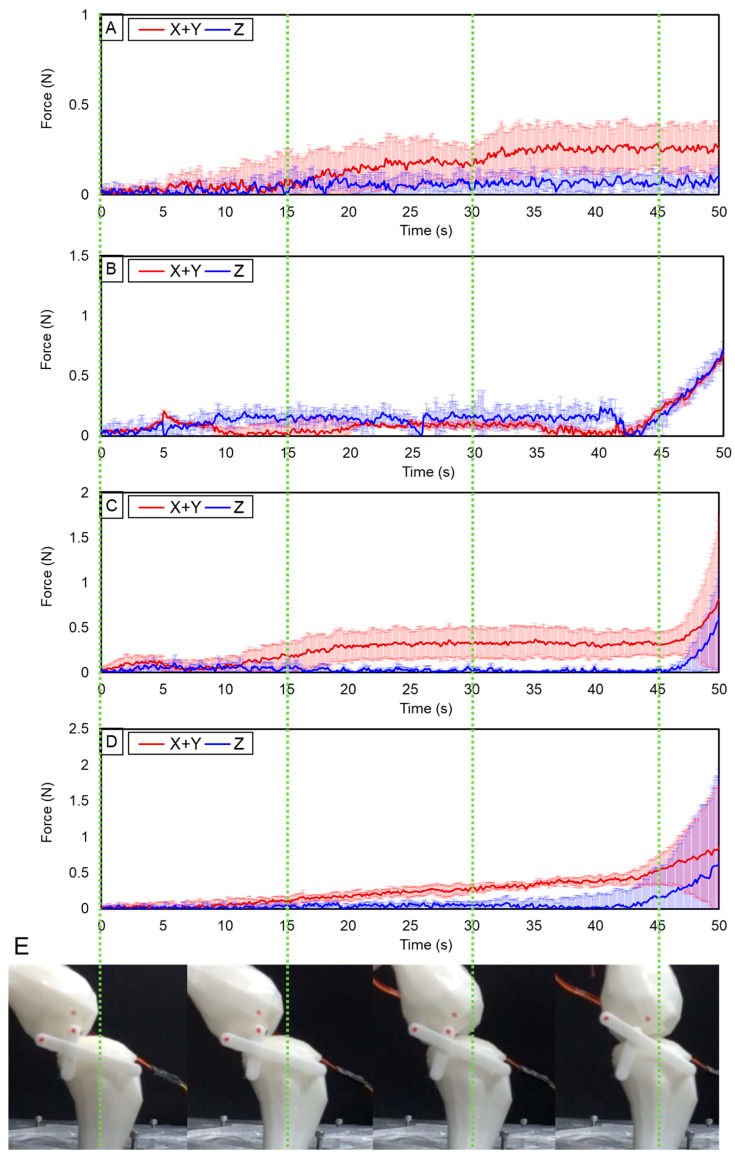
(**A**) Result of measuring the force with a load cell for Level 1, (**B**) Level 2, (**C**) Level 3, and (**D**) Level 4 knee bone. (**E**) Captured movement at 0 s, 15 s, 30 s, and 45 s.

This study revealed a positive correlation between knee load, cartilage thickness, and skeletal geometry. The forms of cartilage and bone vary depending on the severity of OA. The experiment involved affixing cartilage to the simulated skeleton of individuals with OA grades 1 to 4. Subsequently, motion was induced in the skeletal model, and the resulting force during knee movement was quantified. The experimental results demonstrate a positive correlation between the level of OA and the occurrence of peak force, indicating that as the OA level increases, the peak force also increases. Through five repeated experiments, the average value is shown as one line, and the standard deviation is shown in the shadow of the graph. The greatest force measured at OA level 1 was less than 0.5 N. However, at OA level 4, a force of roughly 2.2 N was observed, representing a change of more than fourfold (Figure 6). This enabled the analysis of forces exerted on the femur and tibia, as well as the stress distribution across the knee joint. It revealed patterns indicating that force levels increased in correlation with higher levels of OA.

Increased joint stress is a recognized risk factor for OA development and progression. It can damage cartilage, leading to its breakdown and loss, which are hallmarks of OA [44,45]. Cartilage is sensitive to mechanical forces, which trigger cellular responses that can either promote cartilage health or degradation. High forces can overload chondrocytes (cartilage cells), leading to inflammatory responses and imbalance in cartilage matrix production and breakdown [46]. Increased stress can also affect the underlying subchondral bone, leading to microfractures and changes in bone density, which can further contribute to OA progression [47]. Therefore, reducing joint stress is a key principle of OA treatment. This can be achieved through weight loss, exercise programs aimed at strengthening supporting muscles, and the use of assistive devices like canes or braces [48,49].

During the movement, the load was concentrated in the forward direction. Looking at the following structure of the knee joint, the tibia is supported by the femur, acetabulum, and ligaments and muscles. The tibia measuring the experimental value intersects between the upper part of the femur and the lower part of the acetabulum. Due to this structure, the knee joint mainly moves back and forth. When walking, since the knee is bent and then stretched, there is a tendency to apply a load in the forward direction to the knee joint. Several environmental factors are known to contribute to an increased rate of change in joint cartilage. Repetitive movements, overuse of joints, or sustaining high loads on joints, especially heavy lifting, can increase mechanical stress on joint cartilage [50]. Excess body weight increases the mechanical load on weight-bearing joints like the knees and hips. This additional stress can accelerate cartilage breakdown and exacerbate OA symptoms [51]. It was reflected in the experiment results. Figure 6 reflects the results of this movement well, and the upper and anterior loads gradually become stronger.

In this experiment, since it is a convex on concave situation, the femur in the back comes upward and forward as the knee is stretched. Therefore, it is the same situation as the pressure of the knee joint is applied in the forward direction when walking or running in everyday life. The application of force forward is also related to thigh muscle groups. The square muscle, the femoral biceps, and the femoral biceps support and move the knee joints, and these muscles apply pressure forward when extending the thigh. It creates an anterior load on the knee joint as well.

### 3.2. Trajectory of the Knee Model

The knee’s rolling and sliding motion was achieved by employing the four-bar-link mechanism developed according to established specifications, although it is difficult to completely simulate the complex movement of the actual knee joint.

**Figure 7:**
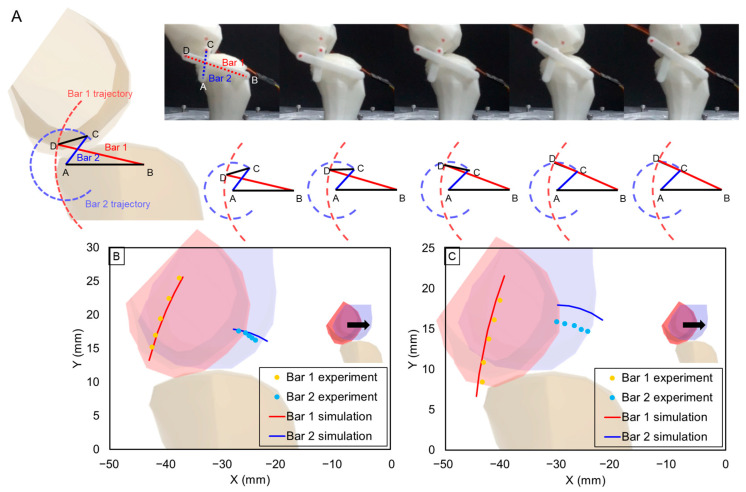
(**A**) is captured movement during experiment. The simulating trajectories are shown. The experimental results of measuring the trajectory when (**B**) is normal and (**C**) is OA level 4.

The four-bar-link mechanism is one of the general mechanisms used to simplify and imitate knee joint movement. The system is characterized by its simplicity and efficiency, which is achieved by a limited number of links and an intuitive joint construction. However, the straightforward link construction might restrict the ability to produce a wide range of turning radii. In the four-bar-link theory, two bars draw a circle based on each midpoint. Figure 7A shows the theoretical four-bar-link movements that occur according to knee joint rotation in chronological order. Figure 7B,C shows the theoretical trajectory derived from the linkages of the four-bar-link and the experimental trajectory of the endpoint of the fabricated link. Figure 7B represents the normal model, while Figure 7C depicts the outcome of implementing the skeletal model of OA level 4. The similarity between the simulated and experimental values of bar 1 was confirmed in both the normal model and the OA level 4 model. However, in the case of bar 2, there was a discrepancy between the theoretical and experimental values in the patient model. Furthermore, in the case of the patient model, it was verified that the trajectory slope of bar1 exhibited a pronounced steepness. In the graph, link 1 indicates an average movement of 38 degrees, whereas link 2 indicates an average movement of 40 degrees.

Although the thigh muscles were not considered in this study, it can be confirmed that the trajectory is drawn from the back to the front because it simulates the ligaments due to the four-bar-link.

## 4. Discussion

The knee movement simulation system visualizes joint function and contact forces by simulating various scenarios and observing their impact on joint mechanics. The force levels showed in this study increased with higher OA levels. This suggests a direct relationship between joint stress and the progression of osteoarthritis. Research suggests a connection between increased joint forces and OA severity, which was also studied in various ways [52,53]. The type of activity and how the force is distributed across the joint can influence the impact on cartilage health. For example, Connelly et al. show that high-impact activities might be more detrimental than low-impact exercises [54]. Understanding this relationship can help in early diagnosis and in slowing down the progression of the disease. Integrating knee movement simulation systems into clinical practice can enhance diagnostic accuracy and personalized treatment plans for knee conditions. Based on the individual’s movement deficits and biomechanical profile, the system can generate personalized rehabilitation exercises and routines, optimizing them. The development of a skeletal model using artificial bone replica can also be used in the development of artificial joints. Artificial knee joints are used in patients who have lost knee function due to knee joint disease or damage. The potential exists for the development of patient-specific prosthetic joints by utilizing skeletal models of patients afflicted with diverse ailments.

Simulating the movement of the knee joint is also helpful in understanding the nature of related diseases such as OA and knee degenerative diseases. Research on knee joints can play an important role in many aspects, such as disease prevention, treatment development, improvement of patient quality of life, improvement of exercise performance, and development of artificial joint technology.

The method used in this study is four-bar-link. The four-bar-link is effective to model the knee joint and simply analyze the force acting on it. In particular, when used with the artificial bone, it is possible to analyze the area under load. Although it has simple and economic advantages, it is difficult to completely imitate the knee because the actual knee joint has a more complex structure and various movements. Since four-bar-link was used, there was insufficient consideration of torque and stress. The four-bar-link model is a rigid model and does not take into account the stress and torque distribution of the actual knee joint. The actual joint has various stresses under various conditions.

Therefore, a more complex mechanism is needed to perfectly simulate the biomechanics of the knee joint. The four-bar-link may be used to mimic some characteristics of the knee joint, but it does not completely simulate all the complexities of the actual knee joint. Since the actual movement and trajectory of the knee joint are determined by various elements and conditions of the human body, it is necessary to present an advanced link through further follow-up studies.

Since the role of ligaments and muscles in human knee joints is important, if an actuator that acts as a muscle instead of a motor is developed and used, it will be possible to simulate the movement of soft knee joints.

## 5. Conclusions

This study implemented the geometry of the knee bone based on the OA level, and confirmed the difference between the osteoclasts and cartilage that changed. Knee movement was implemented through four-bar-link, and the magnitude of the force was also measured when the tibia implemented rolling and sliding on the femur. Furthermore, the trajectory of the skeletal structure during knee flexion was empirically evaluated using a knee simulation system with a four-bar-link.

The research delves into the biomechanics of the knee joint, exploring the stages of OA using the KL grade scale. The force applied to the knee bone varies with the movement of the joint, and studies show distinct characteristics at each stage of OA.

The developed knee simulation system, based on the four-bar-link theory, presents a novel approach to studying OA levels 0 to 4. By measuring force changes under the femur bone during simulated patient exercises, the system provides valuable insights into the knee’s condition. This measurement system has the potential for application in various fields, including the advancement of surgical simulators and the analysis of differences among OA patients.

With the combination of measurement systems and knee simulation, a deeper understanding of knee biomechanics is possible. The development of effective diagnostic and intervention strategies for knee-related conditions can ultimately improve patient outcomes and quality of life. It can be utilized to customize prosthetics to the specific biomechanical needs of individual patients. This personalized approach can significantly improve the success rates of prosthetic surgeries and patient satisfaction. Insights from the study could lead to the development of advanced orthopedic braces and supports which could be designed to better distribute stress across the knee joint, providing relief and support for those with weakened or damaged knees. For individuals at risk of knee joint issues, such as athletes or people with early signs of joint degradation, the findings could guide preventive interventions to maintain joint health and function and potentially delay or avoid the need for prosthetics. The findings from the study can inspire the design of artificial joints and rehabilitation equipment that more closely mimic the natural movement and stress distribution of the human knee especially in the biomimetic field. The study’s insights into the stress patterns of the knee joint can guide material scientists in developing new materials for use in joint replacement and repair, which could be more durable and biocompatible. Understanding the forces in the knee joint can inform more effective rehabilitation and physical therapy strategies tailored to restore and maintain optimal joint function. Furthermore, ongoing research on knee simulation, bio-inspired prosthetics, and tissue engineering can contribute to a deeper understanding of knee structure and function. The study could influence the development of wearable technology that monitors joint stress and movement, providing real-time feedback for injury prevention and rehabilitation. In robotics, the study can inform the design of more sophisticated and efficient robotic limbs that replicate human movement more accurately.

## Figures and Tables

**Table 1:** Material Properties of PLA, EcoFlex, and Human Knee Components [39,40,41,42,43].

Parameter	Modulus Range [GPa]
PLA	0.0015–0.003
EcoFlex	0.00002–0.01
Cortical bone	0.010–0.03
Cancellous bone	0.0005–0.002
Muscle	0.0002–0.001
Cartilage	0.0005–0.02
Tendon	0.1–1
Ligament	0.1–0.7

## Data Availability

Data is contained within the article.
